# Comparison between Tetrel Bonded Complexes Stabilized by σ and π Hole Interactions

**DOI:** 10.3390/molecules23061416

**Published:** 2018-06-11

**Authors:** Wiktor Zierkiewicz, Mariusz Michalczyk, Steve Scheiner

**Affiliations:** 1 Faculty of Chemistry, Wrocław University of Science and Technology, Wybrzeże Wyspiańskiego 27, 50370 Wrocław, Poland; mariusz.michalczyk@pwr.edu.pl; 2 Department of Chemistry and Biochemistry, Utah State University, Logan, UT 84322-0300, USA

**Keywords:** MP2, DFT, NBO, MEP, AIM

## Abstract

The σ-hole tetrel bonds formed by a tetravalent molecule are compared with those involving a π-hole above the tetrel atom in a trivalent bonding situation. The former are modeled by TH_4_, TH_3_F, and TH_2_F_2_ (T = Si, Ge, Sn) and the latter by TH_2_=CH_2_, THF=CH_2_, and TF_2_=CH_2_, all paired with NH_3_ as Lewis base. The latter π-bonded complexes are considerably more strongly bound, despite the near equivalence of the σ and π-hole intensities. The larger binding energies of the π-dimers are attributed to greater electrostatic attraction and orbital interaction. Each progressive replacement of H by F increases the strength of the tetrel bond, whether σ or π. The magnitudes of the maxima of the molecular electrostatic potential in the two types of systems are not good indicators of either the interaction energy or even the full Coulombic energy. The geometry of the Lewis acid is significantly distorted by the formation of the dimer, more so in the case of the σ-bonded complexes, and this deformation intensifies the σ and π holes.

## 1. Introduction

Recent years have seen a veritable explosion of research into noncovalent interactions that are analogous to the H-bond. The proton acting as a bridge between the two subunits in the H-bond can be replaced by any of a number of more electronegative atoms, without loss of binding strength. Depending upon the chemical family to which this bridging atom belongs, these noncovalent bonds have been denoted as halogen, chalcogen, and pnicogen bonds [1,2,3,4,5,6,7,8,9,10]. Data has also accumulated that this sort of bonding can also involve the inert gas atoms in aerogen bonds [11] and even the coinage metal atoms in so-called regium bonds [12]. All of these interactions have a number of features in common. Asymmetrical distribution of electron density around the bridging atom typically leads to one or more σ-hole [13,14,15,16,17,18,19,20,21,22,23,24,25] of positive electrostatic potential. Each such σ-hole is situated directly opposite a covalent bond involving the atom of interest, and can attract a nucleophile. To this Coulombic attraction is added other attractive forces identified with charge transfer, polarization, and dispersion.

Another of this set of noncovalent bonds which has begun to garner widespread attention is the tetrel bond, in which the bridging atom belongs to the C/Si Group 14 of the periodic table. Tetrel bonds play an essential role in numerous processes, as for instance the first stages of S_N_2 reactions which are important in organic synthesis [26]. Other works include a study of the carbon bond as representative of tetrel bonds [27], acetonitrile complexes with tetrahalides [28], examples derived from crystal structures [29], steric crowding in FTR_3_ (T = Si, Ge, Sn, Pb) complexes with various Lewis bases [30], factors controlling the strength of tetrel bonds [31], as well as a recent paper regarding the implications of deformation of the tetrel-containing molecule when paired with ammonia, pyrazine and nitrogen cyanide [32].

As study of these noncovalent bonds progressed it soon became apparent that σ-holes are not the only regions of positive potential that may be present. In cases where the bridging atom lies in a planar (or nearly planar) bonding environment, a positive area can develop above this molecular plane [33]. Like σ-holes, these π-holes serve as sites of attraction for an approaching nucleophile [34,35,36,37,38,39,40,41,42]. There have been a number of studies of noncovalent bonds of various sorts that have examined both σ and π-holes, and more interestingly, comparisons between the two [26,33,43,44,45,46]. Lastly, a valuable supplement to this matter concerning the molecular orbital theory-based description of σ, π and δ holes was introduced by Angarov and Kozuch [47]. It is stated there that many chalcogen and pnicogen bonds should be termed as hybrid σ/π hole interactions rather than simple σ-hole. However, these sorts of comparisons are largely absent in the context of tetrel bonds. Given the importance of tetrel bonds, and the preponderance of molecules in which both σ and π holes may be present, a thorough and comprehensive understanding of the forces that contribute to both, and how they compare with one another, is of paramount importance.

It is to this problem that this work is devoted. Systems are developed in which σ and π tetrel bonding may be directly compared with one another in a controlled fashion by quantum chemical calculations. The molecular electrostatic potential is evaluated for each Lewis acid, which reveals all plausible sites of attachment of a nucleophile, and geometry optimizations reveal which of these sites actually result in an equilibrium dimer. One is able to determine how accurate a measure of the binding strength are the intensities of the σ and π-holes. It is also possible to go one step further and assess whether the hole intensity in and of itself is an accurate indicator of the full Coulombic interaction between the two molecules. Beyond this, how does the latter electrostatic term compare with other attractive forces such as charge transfer and dispersion? Given the prior observation that tetrel bonds lead to sizable geometric deformations of the monomers [32,48], how do such distortions factor into the binding energy of the σ and π tetrel bonds? And as a bottom line, how do the strengths of σ and π tetrel bonds compare with one another?

## 2. Systems and Computational Methods

Tetravalent TH_4-*n*_F*_n_* molecules, with T = Si, Ge, and Sn, were taken as systems which contain σ-holes of varying strength. As a point of comparison, TH_2-*n*_F*_n_*=CH_2_ molecules place the T atom in a planar trivalent bonding situation, which can be expected to contain π-holes above the T atom. One can adjust the number *n* of electron-withdrawing F atoms in each molecule so as to modulate the strength of these two sorts of holes, and still facilitate a fair comparison. It is also possible to assess how sensitive the findings might be to the identity of the particular tetrel atom by comparing Si with Ge and Sn. NH_3_ was taken as the universal electron donor, due first to the ready availability of its lone electron pair. The presence of only one such pair, coupled with the small size of this molecule, allows an unambiguous evaluation of the properties of the tetrel bond, minimizing any complicating secondary interactions.

All geometries were optimized at the MP2 level in conjunction with the aug-cc-pVDZ basis set [49,50]. For the Sn atom, the aug-cc-pVDZ-PP basis set from the EMSL library was applied for the purpose of including relativistic effects [51,52]. All complexes were characterized as minima by frequency analysis calculations. The interaction energies of the complexes were evaluated as the difference in energy between the dimer and the sum of the two monomers, frozen in the same geometry as in the dimer, then corrected for basis set superposition error (BSSE) by the standard counterpoise procedure [53]. The deformation energies of the two subunits were assessed as the difference in electronic energy between each unit within the geometry of the complex and that of the fully optimized isolated molecule. Computations were carried out with the Gaussian 09 suite of programs [54]. Energy decomposition analysis (EDA) was performed at the BLYP-D3(BJ)/ZORA/TZ2P level using the ADF program [55,56,57]. The molecular electrostatic potentials (MEPs) of the isolated monomers were evaluated on the electron density isosurface of ρ = 0.001 au at the MP2/aug-cc-pVDZ level, and its extrema were determined using the WFA-SAS program [58]. MP2 electron densities were analyzed via AIM in order to identify the bond critical points (BCPs) [59] and to evaluate their properties. In order to incorporate electron correlation into the NBO analysis of interorbital electron transfer, the BLYP-D3(BJ) functional was applied within the context of the def2TZVPP basis set via the GenNBO program [60]. 

## 3. Results and Discussion

### 3.1. Electrostatic Potentials of Isolated Molecules 

**Figure 1:**
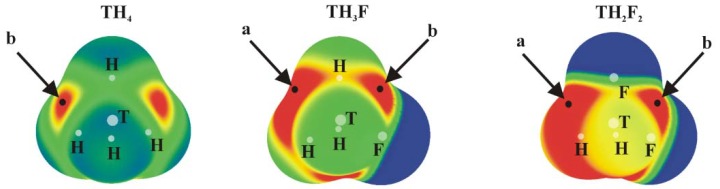
MEPs of TH_4_, TH_3_F and TH_2_F_2_ (T = Si, Ge or Sn) computed on the 0.001 au isodensity surface at the MP2/aug-cc-pVDZ-PP level. Colour ranges, in kcal/mol, are: red greater than 15, yellow between 8 and 15, green between 0 and 8, blue below 0 kcal/mol. The letters a and b mean different types of V_s,max._

**Figure 2:**
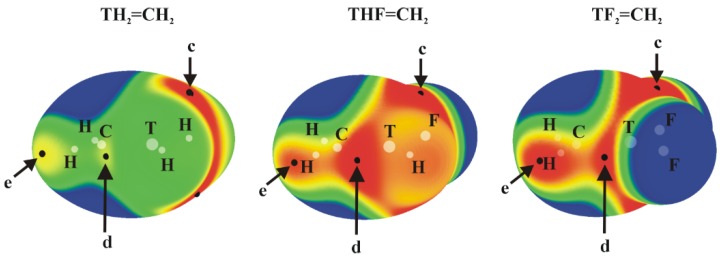
MEPs of TH_2-*n*_F*_n_*=CH_2_ isolated molecules, computed on the 0.001 au isodensity surface at the MP2/aug-cc-pVDZ-PP level. Colour ranges, in kcal/mol, are: red greater than 15, yellow between 8 and 15, green between 0 and 8, blue below 0 kcal/mol. The letters c, d and e mean different types of V_s, max_.

The molecular electrostatic potential (MEP) of the tetravalent, approximately tetrahedral TH_4_, TH_3_F and TH_2_F_2_ (T = Si, Ge or Sn) isolated molecules are displayed in Figure 1; analogous MEPs are shown in Figure 2 for the trivalent TH_2-*n*_F*_n_*=CH_2_ analogues which are roughly planar. 

Positive values of the MEP are denoted in red, while blue represents negative regions. Each of the tetrahedral molecules in Figure 1 contains four σ-holes lying on the extension of each of the four covalent bonds. Due to its symmetry, all four of these MEP maxima are equivalent in TH_4_. There are two types of maxima in the fluorosubstituted species: those opposite F are labeled a, and the b designation is applied to those opposite a H atom. The values of these maxima are collected in Table 1, where it is immediately obvious that a σ-holes opposite F atoms are more intense than their b analogues opposite the H atom. This pattern is consonant with the much greater electronegativity of F; the ratio of a/b values of V_s,max_ varies between 1.4 and 1.8, and their numerical values are consistent with previous studies [24]. Another expected pattern evident in Table 1 is the increase in V_s,max_ as progressively more F atoms are added to the molecule. One normally expects the hole to intensify as the tetrel atom is enlarged. While Sn certainly corresponds to the largest values of V_s,max_, Si and Ge are less distinct from one another.

The planar TH_2-*n*_F*_n_*=CH_2_ molecules contain three primary types of MEP maximum as shown in Figure 2 (their values are given in Table 2). The first, and generally the most intense, is labeled c and occurs roughly above (and below) the T atom, skewed away from the C atom by a certain amount. Maximum d lies in the molecular plane, in a position corresponding roughly to the C=T bond midpoint, approximately on an extension of the T–H or T–F covalent bond.

In most cases, with the sole exceptions of GeHF=CH_2_, and SnHF=CH_2_, maximum c is considerably more positive than is d (see below for further discussion). The last maximum e is associated with the two CH_2_ protons. This position would be pertinent to the formation of any possible CH···N H-bonds with an approaching NH_3_ nucleophile. (Several other maxima appear in some of these molecules but are much weaker in intensity.) Focusing on maximum c, the site of the π-hole, one sees a clear intensification as H atoms are replaced by F. On the other hand, the expected trend of growing intensity with tetrel atom size is violated. Although Sn does indeed produce the largest π-holes, Si exceeds its larger Ge congener. The d patterns are more consistent with expectations, with the caveat that the addition of the second F atom reduces V_s,max_. This lowering is sensible because the proximity of the very electronegative F atom to the hole would mitigate against its positive value. It might be noted here that several of the molecules in Figure 2 are not strictly planar. This point will be discussed in greater detail below.

Finally, with respect to the ammonia molecule, the value of V_s, min_ on the N atom at its lone pair position is −37.7 kcal/mol. Based on the positions and intensities of the various σ-holes, one would anticipate that a nucleophile such as NH_3_ would be attracted to the a maximum, directly opposite the F atom if one is present, and that the strongest tetrel bonds would occur for T=Sn, followed by Ge and then by Si; TH_2_F_2_ ought to engage in a slightly stronger bond than would TH_3_F.

### 3.2. σ-Hole Bonded Dimers

**Figure 3:**
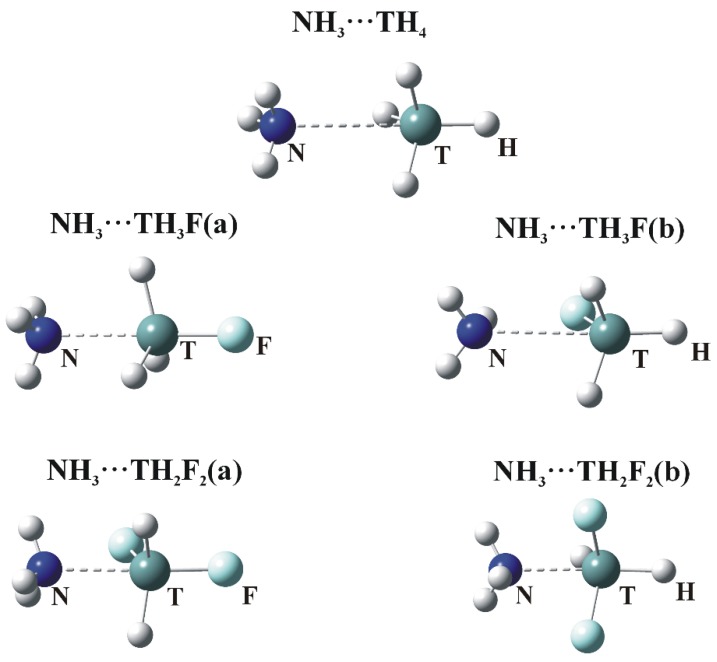
Optimized structures of σ-hole bonded tetrel complexes. (**a**) and (**b**) refer to σ-hole positions in Figure 1 (N—dark blue, T—green, H—white, F—Light blue).

The optimized geometries in which NH_3_ engages with the σ-holes of the tetravalent TH_4_, TH_3_F and TH_2_F_2_ molecules are illustrated in Figure 3. Consistent with the labeling in Figure 1a,b designate whether the N is located opposite the F or H atom, respectively. The interaction energies (E_int_), corrected for BSSE, are collected in Table 3, along with the deformation energies (E_def_) of the subunits as well as selected intermolecular geometrical parameters.

The presence of a tetrel bond is signaled first by the intermolecular R(N∙∙∙T) distance which is smaller than the sum of the corresponding van der Waals radii. (This sum is equal to 3.85, 3.95 and 4.08 Å for Si, Ge and Sn, respectively.) The N atom lies very nearly directly opposite the F atom of the Lewis acid in the a dimers. The θ(R–T∙∙∙N) angle in the last column of Table 3 is 180°, with the exception of TH_2_F_2_. Larger deviations of the θ(HT∙∙∙N) angles from linearity are observed for the b complexes. These nonlinearities are due to attractive forces between the F and H atoms of the Lewis acid and base, respectively.

The interaction energies vary between less than 2 kcal/mol for the TH_4_ molecules to as much as 20 kcal/mol for the difluorinated Lewis acids. The patterns match those of V_s, max_ in Table 1, although imperfectly. In the first place, a dimers with the base opposite F are more strongly bound than b complexes opposite H, but this trend is reversed for GeH_2_F_2_ and SnH_2_F_2_. Whether a or b type, E_int_ rises in the order Si~Ge < Sn, and also increases as more F atoms are added to the acid.

In order to more fully understand the nature of the tetrel bond, and the effects that factor into it, one must first recognize that the formation of such a bond relies on a certain amount of distortion of the monomer geometry. The crowded nature of the tetravalent bonding surrounding the tetrel atom impedes the approach of a nucleophile. Three of the substituents must be peeled back away from this nucleophile to facilitate its approach, which in turn produces a certain amount of deformation energy within the molecule. The magnitude of this deformation energy is listed in Table 3 as E_def_ A for the acid. The NH_3_ molecule need undergo only very little internal deformation so E_def_ B is quite small. E_def_ A is very small for the unsubstituted TH_4_ molecules, not surprising in view of the long intermolecular separations of more than 3 Å. Monofluorination brings the N in much closer, to about 2.6 Å for the a dimers, and the deformation energies are thus larger, nearly 2 kcal/mol. The intermolecular distance is shorter after difluorination and E_def_ A is correspondingly larger, 4–5 kcal/mol. Note that some of the b dimers have an even closer approach, and thus a correspondingly higher deformation energy.

These energies can be correlated to the geometrical changes within the monomers. Summation of the three θ(R_1_TR_2_) angles of the R substituents that come into contact with the nucleophile offers a convenient measure of these distortions. On one extreme, in a fully tetrahedral environment, this sum would be equal to three times 109.5° or 328.5°, which would change to 360° if these three substituents peel back to lie in a plane in a bipyramidal arrangement. This measure of the geometry is listed in Table 4 along with the amount it changes as a result of complexation with NH_3_. Note that there is a very strong correlation between the latter change and deformation energy E_def_ A in Table 3. In fact, the correlation coefficient is 0.999. In either case, the quantity is larger for b than for the a complex for Ge and Sn.

One would expect that the MEPs of these molecules would likewise be altered by the geometrical distortions accompanying dimerization. The effect of the deformation upon the value of V_s, max_ is reported in the last three columns of Table 4 where it may be seen that the partial planarization yields fairly large increases in the MEP maximum, as much as 35 kcal/mol. On a percentage basis, these increases vary from 28% to a near doubling. Note also that the deformation-induced V_s, max_ increase is especially large for the b dimers of Ge and Sn. And it is in just these complexes that one sees an anomalously large interaction energy. On the other hand, it is not just the b geometries for which V_s, max_ grows upon deformation.

The MEP maximum rises also in the a structures, albeit by not as much in the Ge and Sn cases. As a net result, V_s,max_ is larger for a than for b in all of the complexes in Table 4, so one cannot explain the larger interaction energies for the latter solely in terms of MEP. There are of course other aspects of the interaction besides electrostatic attraction. Table 5 presents other components based on an EDA analysis, viz. orbital interaction E_oi_ and dispersion E_disp_. E_elec_ contributes a fairly consistent 52–65% of the total attractive force, differing little between a and b structures. Dispersion makes a smaller contribution, especially in the more strongly bound dimers where it amounts to only about 5%. The orbital interaction term is perhaps more interesting, particularly for the TH_2_F_2_ systems. Parallel to the full E_def_, E_oi_ is larger for the b dimers than for a for both T=Ge and Sn, but the reverse is true for T=Si. It would thus appear that a large part of this pattern can be traced to orbital interactions.

This supposition is confirmed by NBO analysis of the charge transfer. Table S1 demonstrates that two measures of charge transfer conform to the trends listed above. The total intermolecular charge transfer CT is computed as the sum of atomic charge on either monomer. ΣE(2) represents the energetic consequence of transfers from particular molecular orbitals, in this case from the N lone pair to the four antibonding σ*(T–R) orbitals. Both of these parameters are larger for the b than for the a dimer for Ge and Sn, but smaller for Si. And furthermore, they are also larger for a than for b for all the monofluorinated TH_3_F molecules, as was the case for the full interaction energy. 

An alternate means of analyzing the molecular interactions derives from AIM treatment of the topology of the total electron density. Diagrams of the various dimers are provided in Figure S1 for the illustrative Ge set of dimers where small green dots indicate the position of bond critical points. The density, density Laplacian, and total electron energy at the intermolecular bond critical points are collected in Table S2. It might first be noted that there are certain anomalies in this data. In addition to the expected T···N bond paths, there are a number of bond paths placed by AIM between N and certain F atoms of the Lewis acid. Such bonds are reported only for the b type dimers, but not in all cases. The presence of a true N···F bond would contribute to the stability of these geometries. In one case, SiH_2_F_2_(b), a bond path connects N with one of the H atoms of the Lewis acid. Indeed in this case, AIM does not provide evidence of a T···N tetrel bond at all. Dispensing with these anomalies, there are patterns in the AIM data that are consistent with the full energetics. The AIM measures of the Ge···N and Sn···N tetrel bonds in TH_2_F_2_(b) are larger than those for the a analogue, while the opposite may be said for all three TH_3_F dimers.

In summary, the σ-hole directly opposite the F atom is consistently much more positive than one opposite H. Nonetheless, due to a combination of factors, that include deformation-induced intensification, and a greater degree of charge transfer, the latter position becomes competitive with the former as a site for tetrel bonding, and can even surpass the location opposite F as a preferred binding site in certain cases.

### 3.3. π-Hole Bonded Complexes

**Figure 4:**
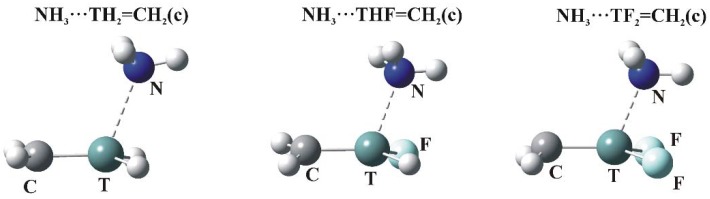
Optimized structures of π-hole bonded tetrel complexes (N—dark blue, T—green, H—white, F—light blue).

As indicated in Figure 2, the MEPs of the planar TH_2-*n*_F*_n_*=CH_2_ molecules have maxima (c) above the molecular plane, in the plane near the C=T midpoint (d), and (e) associated with the CH_2_ protons. The c regions represent the π-hole above the T atom so are the focus of the calculations. The structures of the relevant complexes with NH_3_ are illustrated in Figure 4, and their energetics and geometric details reported in Table 6. 

As in the σ-hole complexes, all T∙∙∙N distances are shorter than the sum of the van der Waals radii of the corresponding atoms. The θ(R–T∙∙∙N) angles are all greater than 90°, reflecting the position of the π-hole maximum. E_int_ varies from a minimum value of 3.7 kcal/mol all the way up to nearly 30 kcal/mol. Just as in the case of V_s,max_ for these π-holes, E_int_ increases steadily as H atoms are replaced by F, with large increments in both quantities associated with each such substitution. 

As in the case of the tetravalent σ-hole complexes described above, formation of the π-hole dimers also impose a certain geometric distortion into the monomers. The deformation energies listed in Table 6 are not insignificant, particularly for the mono and difluorinated species for which E_def_ A varies between 5 and 8 kcal/mol. In this same vein, the various TH_2-*n*_F*_n_*=CH_2_ monomers are not all fully planar and become even less so upon formation of the π-hole dimers. It is a matter of some interest how the interactions might be affected if these molecules were forced to be fully planar within the context of the dimer. Comparison of the second and third columns of Table 6 reveals that such a restriction would severely diminish the interaction energy. This reduction varies from only 1 kcal/mol for GeH_2_=CH_2_ and SnH_2_=CH_2_, but can be as large as 12 kcal/mol for some of the fluorinated species. As a rule of thumb, the various fluorinated Lewis acids lose roughly half of their interaction energy if forced into a planar conformation. But at the same time, it should be stressed that even these reduced interaction energies, in the framework of enforced planarity, still exceed those of the σ-hole dimers in Table 3. The EDA interaction energy contributions of the π-dimers are listed in Table 7.

As in the σ-hole dimers, electrostatics contribute roughly 58–64% of the total attractive interaction. Dispersion is considerably smaller in the π complexes, less than 5%. Orbital interactions account for the difference, making up some 32–40%, as compared to roughly 30% for the σ-dimers. Perhaps more revealing are the absolute values of these components. Both the electrostatic and orbital interaction energies are much larger in magnitude for the π-dimers in Table 7 than for the σ-complexes in Table 5. For example, E_elec_ for the three TH_4_ complexes vary between 3.5 and 8.1 kcal/mol, whereas the analogous values for the corresponding TH_2_=CH_2_ systems lie in the 35.2–64.4 kcal/mol range. The monofluorinated σ dimers cover the 21.3–27.9 range, which is greatly exceeded by the 65.9−86.8 kcal/mol range for the corresponding π-dimers. The same sort of enlargement of the π vs σ complexes is observed in the orbital interaction energies. It is only the dispersion component which is quite similar for the two types of complexes. (This similarity may be due to the use of the Grimme empirical correction, which is not sensitive to the variation of the wave function [61].)

The enlarged contribution from orbital interactions is verified by NBO analysis. As reported in Table S3, the total charge transfer is quite substantial, varying between 113 and 197 me, larger than the same quantities observed for the σ-hole dimers in Table S1. The same amplification applies to the sum of E(2) interorbital transfers, which reach up to nearly 80 kcal/mol in some cases. The magnitudes of these quantities do not closely match the interaction energies. For example, the charge transfers are greatest for Si, as compared to Ge and Sn although the dimers involving Si are not the most strongly bound.

Unlike the σ-hole dimers, the AIM molecular diagrams indicate only a single intermolecular bond path, which corresponds to the T∙∙∙N tetrel bond, as illustrated in Figure S2. The numerical values of the properties of each bond critical point are displayed in Table S5. Like the interaction energies in Table 6, each successive replacement of H by F adds an increment. The comparisons between the three tetrel atoms are, however, not as clear. Taking the three THF=CH_2_ acids as an example, Ge presents the weakest dimer, whereas it shows the largest ρ_BCP_ and H. Comparisons show that the AIM indicators of tetrel bond strength are considerably larger for the π than for the σ-hole tetrel bonds, consistent with the energetic data.

It was pointed out above that the tetravalent TR_4_ molecules undergo significant distortion upon complexation with NH_3_, which in turn enlarges their σ-hole. Table 8 compiles the same sort of data for the π-bonding TR_2_=CH_2_ molecules where the deformation from planarity about both the C and the T atoms are measured by the deviation from 360° of the sum of the three bond angles in which they engage. As may be seen from the second column in Table 8 this nonplanarity only occurs for the difluorinated GeF_2_=CH_2_ and SnF_2_=CH_2_ monomers, and is more exaggerated for the C atom. However, all species become significantly nonplanar in the π-bonded dimers. These deformations about the C atom are fairly small, and only occur for fluorinated species, obeying the T = Si < Ge < Sn pattern. Perhaps more to the point of the interaction of NH_3_ with the T atom, these nonplanar deformations are fairly small, less than 10°.

Contrary to the C deformations, the T nonplanarities follow the opposite Si > Ge > Sn pattern. (It is interesting that the SnF_2_=CH_2_ molecule actually becomes more planar about the Sn atom upon complexation.) In summary, the geometrical distortions induced by π-tetrel bonding are less severe than in the σ-bonded cases, where the deformation measures ranged all the way up to nearly 30°. As in the case of the σ-bonded systems, the deformations of the π-bonding TR_2_=CH_2_ molecules also raise the value of V_s, max_, as is evident in Table 9.

This increase is quite small for TH_2_=CH_2_ but grows as F substituents are added. Just as the trivalent molecules undergo larger geometrical perturbations than do their tetravalent sisters, so too are the π-hole enhancements smaller than those observed in the σ-holes.

### 3.4. Other Geometries

In addition to the c maximum in the MEP of the planar Lewis acids, there is also a d maximum located in the approximate molecular plane, in the vicinity of the T=C midpoint, as detailed in Figure 2. However, optimization of the dimer geometry does not necessarily lead to a minimum in the potential energy surface with the NH_3_ in this position. It is only for the monosubstituted THF=CH_2_ molecule that such a configuration represents a minimum. In some sense this structure resembles a σ-hole dimer, with N situated directly opposite the F atom, rather than a π-dimer. The AIM molecular diagram confirms this to be a T∙∙∙N tetrel bond for Ge and Sn although the bond path for the former is much more curved than is usually the case, as illustrated in Figure S3. But it must be added that this tetrel bond vanishes for the Si system in Figure S3a, leaving only two weak H∙∙∙N interactions, whose ρ and ∇^2^ρ values just barely meet the criteria of hydrogen bonds. 

As may be seen in Table S5, these d dimers are also more weakly bound than the c π-dimers: the former span an E_int_ range between 2.5 and 8.9 kcal/mol, in comparison to the 14.1–19.4 kcal/mol range of the latter. This comparative weakness is in contrast to the values of V_s,max_ in Table 2, for which the d maxima are comparable to, and even exceed the c values. The weaker nature of the d minima extends beyond energetics, encompassing also longer N∙∙∙T distances, and lower E(2) energies, charge transfer, and electronic properties of the BCPs as well, with details contained in Tables S6 and S7. Given the values of V_s, max_ in Table 2, it is perhaps not entirely surprising that it is only the THF=CH_2_ unit that engages in this d bonding. More specifically, the c maximum is much larger than d for both TH_2_=CH_2_ and TF_2_=CH_2_; it is only the monofluorinated species for which the two maxima have comparable values. The EDA results obtained for d complexes are provided in Table S8. As in their analogous c complexes, electrostatic energy contributes about 52–64% of the total attractive interaction, while dispersion is considerably larger, from 9 to 27% in SnHF=CH_2_ and SiHF=CH_2_, respectively. Therefore, the largest contribution of E_disp_ is for the least stable d complex. Orbital interactions in these complexes account for about 23% (average value) which is smaller than those in their stronger c cousins (average value of 37%).

In addition to the dimer geometries described above there were a number of secondary minima, all quite a bit weaker than those described above, none with E_int_ larger than 2 kcal/mol. These weak secondary minima are displayed in Table S9 for the tetrahedral TH_4_, TH_3_F and TH_2_F_2_ molecules, along with their calculated properties. Most dimers are held together by weak H-bonds, and none show any evidence of containing any sort of tetrel bond. Table S10 contains the analogous secondary minima for the planar Lewis acids. Again the primary attractive forces are weak H-bonds and the total interaction energies are rather small.

### 3.5. Discussion

Although the tetrel bond has not been studied as intensively as some of its cousins, e.g., the H-bond or halogen bond, there are nevertheless some prior data that offer points of comparison and context with our own results. The study of complexes of TH_4_ and its mono, tri, and tetrafluorinated derivatives with ammonia (T=Si, Ge, Sn) [31] led to similar conclusions for this different subset of systems. Comparison between intensities of σ-holes exhibits strong similarities and the same trends as those examined here. This earlier work had shown how incorporating monomer deformation energies into the full energetics can lead to somewhat different patterns than the interactions between pre-deformed subunits. Another recent study [62] places the same σ-hole donors in complexes with various π-electron systems acting as Lewis bases. The same Si < Ge < Sn pattern was found there as for the weaker b complexes above, somewhat different than for the more strongly bound a complexes. This work also noted that geometry deformation of the Lewis acid can be negligible, but becomes important for the stronger complexes. Decomposition of interaction energies revealed that the complexes are electrostatically driven and dispersion becomes significant only when the complexes are exceptionally weakly bonded. The vital role of the Pauli repulsion which exceeded the absolute value of the electrostatic component was also noticed. Our results are consistent with these observations. One factor driving the small values of dispersion energy may be the small size of the base, including only a single non-hydrogen atom.

There have been a number of prior studies comparing σ- and π-hole bonded systems. Li’s group [35] paired F_2_C=CFTF_3_ with three Lewis bases including formaldehyde, water, and ammonia, and found π-hole bonded complexes were generally preferred for T=C but the opposite for Si and Ge. With particular respect to NH_3_, the bonding grew in strength as the tetrel atom became larger for both σ- and π-hole complexes, in partial agreement with our results which showed some deviations from this pattern. The interaction energies correlated with the σ-hole intensity of T which was, in turn, strongly associated with the hybridization of C atoms in the order sp^3^ < sp^2^ < sp. A recent [26] perspective article indicates the dominating influence of electrostatic and dispersive terms in both weak σ- and π-hole dimers, in complexes whose deformation energies are close to 0, which was confirmed by Xu et al. [33] based on TH_3_F (T=C and Si) complexes with pyrazine and 1,4-dicyanobenzene. As in the current work, the π-complexes were more stable than their σ counterparts in terms of larger interaction energy, also exhibiting shorter binding distance, greater electron density at BCPs, and larger CT. Also consistent with the data reported above was the distribution of attractive and repulsive components of the interaction, and the consistency with the magnitudes of MEP maxima. Distinctions arise on shifting from tetrel to aerogen atoms. In our own earlier study of aerogen bonds formed between AeOF_2_ (Ae = Kr, Xe) and diazines [43], the σ-hole bonded complexes were considerably stronger than their π-hole analogues.

In the context of the replacement of H atoms by the much more electronegative F, it is typically observed that the interaction grows stronger with each such substitution. For example, early work suggested that tetrafluorosilane was bound to ammonia more tightly than unsubstituted silane [63]. This conclusion was confirmed in later calculations confined to Si [64,65] as well as in the other works that extended to complexes containing heavier tetrel atoms [31,66], and is consistent with our own findings above.

It has been shown in the literature that there are systems where the intensities of the MEP maxima or minima are not necessarily well correlated with interaction energies [32,67,68]. For instance, in the tetrel-bonded complexes of formamidine with TH_3_F (T = C, Si, Ge, and Sn) the interaction energy increases in the order C < Ge < Si < Sn, inconsistent with the magnitude of the σ-hole on the T atom [68]. A similar pattern was found in our current work for the σ-hole bonded (a) dimers. In a recent work [32], a series of complexes pairing Lewis acids TF_4_ or ZF_5_ (T = Si, Ge, Sn and Z = P, As, Sb) with Lewis bases NH_3_, pyrazine, and HCN, the tetrel molecules TF_4_ have a considerably larger (more than 10 kcal/mol) value of V_s,max_ than their corresponding pnicogen ZF_5_ cousin, but nonetheless smaller interaction energy. Moreover, another inconsistency was observed with respect to V_s,min_ which is more negative for NCH than for pyrazine, but the latter complexes investigated were more strongly bound. Similar discrepancies arise in halogen bonded complexes involving chlorinated and methylated amines [67].

The issue of geometrical deformations of the monomers and their impact on tetrel-bonded complexes has been described recently in a few papers [47,69]. In our own latest work devoted to implications of monomer deformation upon tetrel and pnicogen bonds [32] it was shown that complexation can cause monomer deformation which results in a multifold increase in the intensity of V_s,max_, which in turn amplifies the magnitude of the interaction energy.

## 4. Conclusions

In conclusion, the π-complexes formed above the plane of the TR_2_=CH_2_ molecules are more strongly bound than are their quasi-tetrahedral TR_4_ σ congeners, given the same degree of fluorosubstitution. Starting with the unsubstituted species, the interaction energies of TH_2_=CH_2_ vary between 3.7 and 7.8 kcal/mol, considerably larger in magnitude than the 1.6–2.8 kcal/mol range for the σ-bonded TH_4_ species. In the difluorinated sets, the ranges of binding energies of TF_2_=CH_2_ and TF_2_H_2_ are respectively 27.3–29.3 and 10.4–15.3 kcal/mol. This distinction cannot be attributed to the intensity of the π and σ-holes in the MEPs, as they are roughly comparable, and indeed the σ-holes tend to be a bit more intense. In fact, the latter σ-holes grow even larger when the TR_4_ molecules deform into the geometries they adopt within their complex with NH_3_. Contrary to the general similarity between the intensities of the σ and π-holes, the full evaluation of the electrostatic interaction reveals a much greater Coulombic attraction for the π-dimers, coupled with an enlarged orbital interaction energy. It should be emphasized that the stronger binding in the π-complexes cannot be attributed to any geometrical distortions undergone by these pseudoplanar molecules. In the first place, their geometrical deformation upon dimerization is less than that of their tetravalent analogues. And even when these TR_2_=CH_2_ Lewis acid molecules are forced into a fully planar internal geometry, their interaction energy with NH_3_ remains larger than their σ-hole TR_4_ counterparts, even if the latter are permitted to deform within the dimer.

## Figures and Tables

**Table 1:** Values of two maxima in the MEPs (V_s,max_, kcal/mol) of tetravalent σ-hole donors at the MP2/aug-cc-pVDZ-PP level of theory.

T	V_s, max_^a^	TH_4_	TH_3_F	TH_2_F_2_
Si	a	-	41.8	43.9
	b	19.8	26.4	31.1
Ge	a	-	46.0	49.8
	b	18.4	25.3	30.4
Sn	a	-	54.8	59.6
	b	25.0	31.7	37.8

^a^ a and b maxima lie respectively on the extensions of T–F and T–H bonds (see Figure 1).

**Table 2:** Values of maxima in the MEPs (V_s,max_, kcal/mol) of TR_2_=CH_2_ π-hole donors, at the MP2/aug-cc-pVDZ-PP level of theory.

T	V_s, max_^a^	TH_2_=CH_2_	THF=CH_2_	TF_2_=CH_2_
Si	c	21.0	32.4	48.8
	d	10.1	23.9	19.5
	e	12.1	16.3	18.8
Ge	c	19.4	29.8	44.8
	d	10.8	32.5	27.0
	e	12.2	19.2	24.0
Sn^b^	c	24.0	34.8	53.3
	d	14.8	43.6	37.3
	e	11.1	19.5	25.1

^a^ Locations of the maxima are displayed in Figure 2. ^b^ In the SnH_2_=CH_2_ molecule there is another V_s, max_ with a value of 18.1 kcal/mol located on the extension of the C=Sn bond (between two c maxima).

**Table 3:** Interaction energy (E_int_) corrected for BSSE, of indicated Lewis acid with NH_3_ in σ-hole bonded complexes, along with deformation energy (E_def_) of individual subunits, intermolecular distance and angle (energies in kcal/mol, distances in Å, angles in degrees). Data obtained at the MP2 level of theory.

Lewis Acid	E_int_	E_def_ A ^a^	E_def_ B ^b^	R(N∙∙∙T)	θ(R–T∙∙∙N) ^c^
SiH_4_	−1.8	0.14	0	3.232	180
GeH_4_	−1.59	0.11	0	3.332	179.6
SnH_4_	−2.81	0.37	0	3.170	180
SiH_3_F(a)	−7.43	1.93	0	2.557	180
SiH_3_F(b)	−3.24	0.34	0	3.102	174
GeH_3_F(a)	−7.34	1.49	0	2.630	179.9
GeH_3_F(b)	−3.72	0.29	0	3.134	170.5
SnH_3_F(a)	−10.29	1.78	0	2.667	180
SnH_3_F(b)	−7.43	2.23	0.03	2.793	166.2
SiH_2_F_2_(a)	−10.42	5.07	0.02	2.390	177.6
SiH_2_F_2_(b)	−4.12	1.38	0	2.865	175.8
GeH_2_F_2_(a)	−10.84	3.97	0.02	2.458	174.3
GeH_2_F_2_(b)	−11.34	9.14	0.06	2.364	168.2
SnH_2_F_2_(a)	−15.29	3.77	0.04	2.521	169.2
SnH_2_F_2_(b)	−20.07	10.45	0.14	2.374	155.8

^a^ Deformation energy of Lewis acid. ^b^ Deformation energy of Lewis base (NH_3_).^c^ R refers to F or H in complexes (**a**) and (**b**), respectively.

**Table 4:** Planarity measure and MEP maximum of TH_2_F_2_ molecule in its geometry within the monomer and within its complex with NH_3._

	Σθ(R1TR2), degs			V_s, max_, kcal/mol		
	Monomer	Complex	Change	Monomer	Complex	Change
Si a	332.3	350.0	17.7	43.9	60.9	17.0
Si b	324.1	334.3	10.2	31.1	42.9	11.8
Ge a	335.1	351.0	15.9	49.8	63.7	13.9
Ge b	320.7	346.9	26.2	30.4	58.9	28.5
Sn a	337.1	352.9	15.8	59.6	74.0	14.4
Sn b	318.4	347.5	29.1	37.8	72.8	35.0

**Table 5:** EDA/BLYP-D3(BJ)/ZORA/TZ2P decomposition of the interaction energy of σ-hole bonded complexes into Pauli repulsion (E_Pauli_), electrostatic (E_elstat_), orbital interaction (E_oi_) and dispersion (E_disp_) terms. All energies in kcal mol^−1^. The relative values in percent express the contribution of each to the sum of all attractive energy terms.

Lewis Acid	ΔE	E_Pauli_	E_elec_	%	E_oi_	%	E_disp_	%
SiH_4_	−2.12	5.8	−4.2	53	−1.98	25	−1.73	22
GeH_4_	−1.69	5.03	−3.52	52	−1.53	23	−1.67	25
SnH_4_	−3.04	10.55	−8.12	60	−3.18	23	−2.29	17
SiH_3_F(a)	−8.64	29.45	−22.44	59	−12.88	34	−2.77	7
SiH_3_F(b)	−3.66	8.78	−7.43	60	−2.85	23	−2.15	17
GeH_3_F(a)	−7.29	27.28	−21.29	62	−10.59	31	−2.7	8
GeH_3_F(b)	−3.95	9.52	−8.24	61	−2.99	22	−2.24	17
SnH_3_F(a)	−9.92	33.5	−27.86	64	−12.54	29	−3.01	7
SnH_3_F(b)	−7.54	28.65	−23.26	64	−9.85	27	−3.07	8
SiH_2_F_2_(a)	−11.22	48.26	−36	61	−20.34	34	−3.11	5
SiH_2_F_2_(b)	−4.8	16.04	−13.24	64	−4.99	24	−2.6	12
GeH_2_F_2_(a)	−10	45.54	−34.9	63	−17.59	32	−3.05	5
GeH_2_F_2_(b)	−10.53	62.38	−46.24	63	−23.32	32	−3.34	5
SnH_2_F_2_(a)	−14.16	50.53	−41.9	65	−19.42	30	−3.36	5
SnH_2_F_2_(b)	−18.91	75.93	−61.53	65	−29.58	31	−3.73	4

**Table 6:** Interaction energy (E_int_) corrected for BSSE, subunit deformation energy (E_def_), and intermolecular geometrical parameters (energies in kcal/mol, distances in Å, angles in degrees) in π-hole bonded complexes with NH_3_. Data obtained at the MP2 level of theory.

Lewis Acid	E_int_	E_int_ (Planar) ^a^	E_def_ A	E_def_ B	R(N∙∙∙T)	θ(R–T∙∙∙N)
SiH_2_=CH_2_	−7.82	−3.57	2.08	0.10	2.176	113.3
GeH_2_=CH_2_	−3.72	−2.79	0.88	0.04	2.460	112.0
SnH_2_=CH_2_	−5.79	−4.80	0.63	0.05	2.582	104.6
SiHF=CH_2_	−19.64	−8.10	6.29	0.15	2.052	111.9
GeHF=CH_2_	−14.13	−6.71	4.84	0.14	2.184	110.8
SnHF=CH_2_	−19.37	−10.49	6.41	0.18	2.356	100.2
SiF_2_=CH_2_	−28.30	−15.70	5.81	0.15	2.003	116.2
GeF_2_=CH_2_	−27.26	−14.90	8.01	0.16	2.094	111.5
SnF_2_=CH_2_	−29.02	−19.17	6.75	0.21	2.296	106.4

^a^ Lewis acid molecule restrained to planarity.

**Table 7:** EDA/BLYP-D3(BJ)/ZORA/TZ2P decomposition of the interaction energy of π-hole bonded complexes into Pauli repulsion (E_Pauli_), electrostatic (E_elstat_), orbital interaction (E_oi_) and dispersion (E_disp_) terms. All energies in kcal/mol. The relative values in percent express the contribution of each to the sum of all attractive energy terms.

Lewis Acid	E_int_	E_Pauli_	E_elec_	%	E_oi_	%	E_disp_	%
SiH_2_=CH_2_	−9.15	101.19	−64.42	58	−43.04	39	−2.88	3
GeH_2_=CH_2_	−4.24	54.47	−35.18	60	−20.63	35	−2.91	5
SnH_2_=CH_2_	−6.73	54.61	−38.58	63	−19.77	32	−3.00	5
SiHF=CH_2_	−19.87	128.97	−86.78	58	−58.74	40	−3.17	2
GeHF=CH_2_	−12.33	107.09	−72.17	60	−44.06	37	−3.19	3
SnHF=CH_2_	−19.09	84.72	−65.89	63	−34.47	33	−3.46	3
SiF_2_=CH_2_	−27.53	139.3	−97.5	58	−66.02	40	−3.31	2
GeF_2_=CH_2_	−24.73	122.68	−88.6	60	−55.41	38	−3.39	2
SnF_2_=CH_2_	−26.98	88.69	−74.07	64	−38.05	33	−3.55	3

**Table 8:** Planarity measure of TR_2_=CH_2_ molecule in its geometry within the monomer and its π-bonded c complex with NH_3._

	Σθ(R_1_CR_2_), degs			Σθ(R_1_TR_2_), degs		
	Monomer	Complex	Change	Monomer	Complex	Change
SiH_2_=CH_2_	360	359.9	−0.1	359.9	353.9	−6.0
GeH_2_=CH_2_	360	359.6	−0.4	359.9	356.8	−3.1
SnH_2_=CH_2_	360	359.6	−0.4	360	359	−1.0
SiHF=CH_2_	359.9	359.6	−0.3	360	353.3	−6.7
GeHF=CH_2_	360	359	−1.0	360	355.6	−4.4
SnHF=CH_2_	359.8	343.4	−16.4	359.9	359.8	−0.1
SiF_2_=CH_2_	360	359.5	−0.5	360	352.5	−7.5
GeF_2_=CH_2_	353.7	345.6	−8.1	357.9	357	−0.9
SnF_2_=CH_2_	337.5	327.2	−10.3	351.6	359.8	+8.2

**Table 9:** Magnitude of V_s, max_ (kcal/mol) on T atom of isolated TR_2_=CH_2_ molecule and its value when the molecule is distorted to that within the π-bonded c complex.

	Monomer	Complex	Change
SiH_2_=CH_2_	21.0	23.0	2.0
GeH_2_=CH_2_	19.4	20.6	1.2
SnH_2_=CH_2_	24.0	24.4	0.4
SiHF=CH_2_	32.4	39.2	6.8
GeHF=CH_2_	29.8	34.7	4.9
SnHF=CH_2_	34.8	49.1	14.3
SiF_2_=CH_2_	48.8	54.3	5.5
GeF_2_=CH_2_	44.8	58.4	13.6
SnF_2_=CH_2_	53.3	78.5	25.2

## Supplementary Materials

The following are available online, Figure S1: AIM diagrams showing the bond critical points (green dots) in Ge-containing complexes stabilized by σ-hole tetrel bonds, Figure S2: Bond critical points (green dots) in several Ge-containing complexes stabilized by π-hole tetrel bond, Figure S3: AIM molecular diagram of THF=CH_2_/NH_3_ d dimers wherein the base occupies the d maximum of the MEP of the acid, Table S1: NBO values of sum of the E(2) for LP(N)→σ*(T–X), (T= Si, Ge or Sn and X=H or F) orbital interaction and total charge transfer (CT) from NH_3_ to TH_2-n_F_n_ in σ-hole bonded complexes obtained at the BLYP-D3(BJ)/def2-TVZPP level, Table S2: AIM data for σ-hole bonded complexes. Bond critical point (BCP) properties: electron density *ρ*, Laplacian of electron density ∇^2^*ρ* (both in atomic units) and total electron energy (H, kcal mol^−1^). Calculations were performed at the MP2/aug-cc-pVDZ-PP level, Table S3: NBO values of sum of the E(2) for LP(N)→σ*(T–X), (T= Si, Ge or Sn and X=H or F) orbital interaction and total charge transfer (CT) from NH_3_ to TH_2-n_F_n_=CH_2_ in π-hole bonded complexes obtained at the BLYP-D3(BJ)/def2-TVZPP level, Table S4: AIM data for π-hole bonded complexes. Bond critical point (BCP) properties: electron density *ρ*, Laplacian of electron density ∇^2^*ρ* (both in atomic units) and total electron energy (H, kcal mol^−1^). Calculations were performed at the MP2/aug-cc-pVDZ level, Table S5: Geometry and energetics for d complexes, Table S6: NBO properties of d complexes, Table S7: AIM parameters of d complexes, Table S8: EDA/BLYP-D3(BJ)/ZORA/TZ2P decomposition of the interaction energy of π-hole bonded complexes d into Pauli repulsion (E_Pauli_), electrostatic (E_elec_), orbital interaction (E_oi_) and dispersion (E_disp_) terms. All energies in kcal/mol. The relative values in percent express the contribution of each to the sum of all attractive terms, Table S9: Secondary minima for dimers of NH_3_ with σ-hole donors. Data obtained at the MP2/aug-cc-pVDZ-PP level of theory. E_int_ corrected for BSSE (in kcal/mol). Distances are in Å, Table S10: Secondary minima for dimers of NH_3_ with π-hole donors. Data obtained at the MP2/aug-cc-pVDZ-PP level of theory. E_int_ corrected for BSSE (in kcal/mol). Distances are in Å.

## References

[1] Desiraju G.R., Ho P.S., Kloo L., Legon A.C., Marquardt R., Metrangolo P., Politzer P., Resnati G., Rissanen K. (2013). Definition of the halogen bond (IUPAC recommendations 2013). Pure Appl. Chem..

[2] Cavallo G., Metrangolo P., Milani R., Pilati T., Priimagi A., Resnati G., Terraneo G. (2016). The halogen bond. Chem. Rev..

[3] García-Llinás X., Bauzá A., Seth S.K., Frontera A. (2017). Importance of R-CF3⋯O tetrel bonding interactions in biological systems. J. Phys. Chem. A.

[4] Esrafili M.D., Mohammadian-Sabet F. (2016). Homonuclear chalcogen-chalcogen bond interactions in complexes pairing YO3 and YHX molecules (Y=S, Se; X=H, Cl, Br, CCH, NC, OH, OCH_3_): Influence of substitution and cooperativity. Int. J. Quantum Chem..

[5] Bauzá A., Mooibroek T.J., Frontera A. (2016). σ-Hole opposite to a lone pair: unconventional pnicogen bonding interactions between ZF3(Z=N, P, As, and Sb) compounds and several donors. Chem. Phys. Chem..

[6] Bauza A., Alkorta I., Frontera A., Elguero J. (2013). On the reliability of pure and hybrid DFT methods for the evaluation of halogen, chalcogen, and pnicogen bonds involving anionic and neutral electron donors. J. Chem. Theory Comput..

[7] Iwaoka M., Komatsu H., Katsuda T., Tomoda S. (2002). Quantitative evaluation of weak nonbonded Se···F interactions and their remarkable nature as orbital interactions. J. Am. Chem. Soc..

[8] Azofra L.M., Alkorta I., Scheiner S. (2015). Chalcogen bonds in complexes of SOXY (X, y = F, Cl) with nitrogen bases. J. Phys. Chem. A.

[9] Scheiner S. (2011). Effects of multiple substitution upon the P⋯N noncovalent interaction. Chem. Phys..

[10] Kolar M.H., Hobza P. (2016). Computer modeling of halogen bonds and other σ-hole interactions. Chem. Rev..

[11] Bauza A., Frontera A. (2015). Aerogen bonding interaction: a new supramolecular force?. Angew. Chem. Int. Ed..

[12] Stenlid J.H., Johansson A.J., Brinck T. (2018). σ-Holes and σ-lumps direct the Lewis basic and acidic interactions of noble metal nanoparticles: Introducing regium bonds. Phys. Chem. Chem. Phys..

[13] Murray J.S., Lane P., Politzer P. (2009). Expansion of the σ-hole concept. J. Mol. Model..

[14] Murray J.S., Lane P., Clark T., Riley K.E., Politzer P. (2012). σ-Holes, π-holes and electrostatically-driven interactions. J. Mol. Model..

[15] Bundhun A., Ramasami P., Murray J.S., Politzer P. (2013). Trends in σ-hole strengths and interactions of F3MX molecules (M = C, Si, Ge and X = F, Cl, Br, I). J. Mol. Model..

[16] Bauza A., Mooibroek T.J., Frontera A. (2013). Tetrel-bonding interaction: Rediscovered supramolecular force?. Angew. Chem. Int. Ed..

[17] Politzer P., Murray J.S., Clark T. (2013). Halogen bonding and other σ-hole interactions: A perspective. Phys. Chem. Chem. Phys..

[18] Clark T., Hennemann M., Murray J.S., Politzer P. (2007). Halogen bonding: The σ-hole: Proceedings of “Modeling interactions in biomolecules II”, Prague, September 5th-9th, 2005. J. Mol. Model..

[19] Auffinger P., Hays F.A., Westhof E., Ho P.S. (2004). Halogen bonds in biological molecules. Proc. Natl. Acad. Sci. USA.

[20] Clark T. (2013). σ-Holes. WIREs Comput. Mol. Sci..

[21] Politzer P., Lane P., Concha M.C., Ma Y., Murray J.S. (2007). An overview of halogen bonding. J. Mol. Model..

[22] Stone A.J. (2013). Are Halogen Bonded Structures Electrostatically Driven?. J. Am. Chem. Soc..

[23] Politzer P., Murray J.S. (2013). Halogen bonding: An interim discussion. Chem. Phys. Chem..

[24] Eramian H., Tian Y.-H., Fox Z., Beneberu H.Z., Kertesz M. (2013). On the anisotropy of van der waals atomic radii of O, S, Se, F, Cl, Br, and I. J. Phys. Chem. A.

[25] Politzer P., Murray J.S., Clark T. (2010). Halogen bonding: An electrostatically-driven highly directional noncovalent interaction. Phys. Chem. Chem. Phys..

[26] Grabowski S.J. (2017). Hydrogen bonds, and σ-hole and π-hole bonds-mechanisms protecting doublet and octet electron structures. Phys. Chem. Chem. Phys..

[27] Mani D., Arunan E. (2013). The X-C⋯Y (X = O/F, y = O/S/F/Cl/Br/N/P) ‘carbon bond’ and hydrophobic interactions. Phys. Chem. Chem. Phys..

[28] Helminiak H.M., Knauf R.R., Danforth S.J., Phillips J.A. (2014). Structural and energetic properties of acetonitrile-group IV (A & B) halide complexes. J. Phys. Chem. A.

[29] George J., Dronskowski R. (2017). Tetrel bonds in infinite molecular chains by electronic structure theory and their role for crystal stabilization. J. Phys. Chem. A.

[30] Scheiner S. (2018). Steric Crowding in Tetrel Bonds. J. Phys. Chem. A.

[31] Scheiner S. (2017). Systematic elucidation of factors that influence the strength of tetrel bonds. J. Phys. Chem. A.

[32] Zierkiewicz W., Michalczyk M., Scheiner S. (2018). Implications of monomer deformation for tetrel and pnicogen bonds. Phys. Chem. Chem. Phys..

[33] Xu H., Cheng J., Yang X., Liu Z., Li W., Li Q. (2017). Comparison of σ-hole and π-hole tetrel bonds formed by pyrazine and 1,4-dicyanobenzene: The interplay between anion-π and tetrel bonds. Chem. Phys. Chem..

[34] Politzer P., Murray J.S., Scheiner S. (2015). A unified view of halogen bonding, hydrogen bonding and other σ-hole interactions. Noncovalent Forces.

[35] Wenbo D., Xin Y., Jianbo C., Wenzuo L., Qingzhong L. (2018). Comparison for σ-hole and π-hole tetrel-bonded complexes involving F_2_CCFTF_3_ (TC, Si, and Ge): Substitution, hybridization, and solvation effects. J. Fluorine Chem..

[36] Politzer P., Murray J.S., Clark T. (2015). σ-Hole bonding: A physical interpretation. Top. Curr. Chem..

[37] Azofra L.M., Alkorta I., Scheiner S. (2014). Noncovalent interactions in dimers and trimers of SO_3_ and CO. Theor. Chem. Acc..

[38] Bauza A., Ramis R., Frontera A. (2014). A combined theoretical and cambridge structural database study of π-hole pnicogen bonding complexes between electron rich molecules and both nitro compounds and inorganic bromides (YO_2_Br, Y = N, P, and As). J. Phys. Chem. A.

[39] Bauza A., Mooibroek T.J., Frontera A. (2015). Directionality of π-holes in nitro compounds. Chem. Commun..

[40] Sanchez-Sanz G., Trujillo C., Solimannejad M., Alkorta I., Elguero J. (2013). Orthogonal interactions between nitryl derivatives and electron donors: Pnictogen bonds. Phys. Chem. Chem. Phys..

[41] Murray J.S., Lane P., Clark T., Riley K.E., Politzer P. (2012). Σ-holes, π-holes and electrostatically-driven interactions. J. Mol. Model..

[42] Del Bene J.E., Alkorta I., Elguero J. (2013). Characterizing complexes with pnicogen bonds involving sp2 hybridized phosphorus atoms: (H_2_C═PX)_2_ with X = F, Cl, OH, CN, NC, CCH, H, CH_3_, and BH_2_. J. Phys. Chem. A.

[43] Zierkiewicz W., Michalczyk M., Scheiner S. (2018). Aerogen bonds formed between AeOF_2_ (Ae = Kr, Xe) and diazines: comparisons between σ-hole and π-hole complexes. Phys. Chem. Chem. Phys..

[44] Politzer P., Murray J.S. (2018). σ-holes and π-holes: Similarities and differences. J. Comput. Chem..

[45] Zhang Y., Wang D., Wang W. (2018). Beyond the σ-hole and π-hole: The origin of the very large electrophilic regions of fullerenes and carbon nanotubes. Comp. Theor. Chem..

[46] Wei Y., Li Q. (2018). Comparison for σ-hole and π-hole tetrel-bonded complexes involving cyanoacetaldehyde. Mol. Phys..

[47] Angarov V., Kozuch S. (2018). On the σ, π and δ hole interactions: A molecular orbital overview. New J. Chem..

[48] Scilabra G., Terraneo G. (2017). Resnati, Fluorinated elements of Group 15 as pnictogen bond donor sites. J. Fluorine Chem..

[49] Møller C., Plesset M.S. (1934). Note on an approximation treatment for many-electron systems. Phys. Rev..

[50] Dunning T.H. (1989). Gaussian basis sets for use in correlated molecular calculations. I. The atoms boron through neon and hydrogen. J. Chem. Phys..

[51] Peterson K.A. (2003). Systematically convergent basis sets with relativistic pseudopotentials. I. Correlation consistent basis sets for the post-*d* group 13–15 elements. J. Chem. Phys..

[52] Schuchardt K.L., Didier B.T., Elsethagen T., Sun L., Gurumoorthi V., Chase J., Li J., Windus T.L. (2007). Basis Set Exchange: A community database for computational sciences. J. Chem. Inf. Model..

[53] Boys S.F., Bernardi F. (1970). The calculation of small molecular interactions by the differences of separate total energies. Some procedures with reduced errors. Mol. Phys..

[54] Frisch M.J., Trucks G.W., Schlegel H.B., Scuseria G.E., Robb M.A., Cheeseman J.R., Scalmani G., Barone V., Mennucci B., Petersson G.A. (2009). Gaussian 09, Revision E.01.

[55] Te Velde G., Bickelhaupt F.M., Baerends E.J., Fonseca Guerra C., van Gisbergen S.J.A., Snijders J.G., Ziegler T. (2001). Chemistry with ADF. J. Comput. Chem..

[56] Fonseca Guerra C., Snijders J.G., te Velde G., Baerends E.J. (1998). Towards an order-N DFT method. Theor. Chem. Acc..

[57] (2014). ADF 2014.

[58] Bulat F., Toro-Labbe A., Brinck T., Murray J.S., Politzer P. (2010). Quantitative analysis of molecular surfaces: Areas, volumes, electrostatic potentials and average local ionization energies. J. Mol. Model..

[59] Todd A. (2014). Keith AIMAll Version 14.11.23.

[60] Glendening E.D., Landis C.R., Weinhold F. (2013). NBO 6.0: Natural bond orbital analysis program. J. Comput. Chem..

[61] Grimme S., Ehrlich S., Goerigk L. (2011). Effect of the damping function in dispersion corrected density functional theory. J. Comp. Chem..

[62] Grabowski S.J. (2018). Tetrel bonds with π-electrons acting as lewis bases—theoretical results and experimental evidences. Molecules.

[63] Rossi A.R., Jasinski J.M. (1990). Theoretical studies of neutral silane-ammonia adducts. J. M. Chem. Phys. Lett..

[64] Alkorta I., Rozas I., Elguero J. (2001). Molecular complexes between silicon derivatives and electron-rich groups. J. Phys. Chem. A.

[65] Schoeller W.W., Rozhenko A. (2000). Pentacoordination at fluoro-substituted silanes by weak lewis donor addition. Eur. J. Inorg. Chem..

[66] Grabowski S.J. (2014). Tetrel bond–σ-hole bond as a preliminary stage of the S_N_2 reaction. Phys. Chem. Chem. Phys..

[67] Xu H., Cheng J., Yu X., Li Q. (2018). Abnormal tetrel bonds between formamidine and th_3_f: substituent effects. Chem. Sel..

[68] Zierkiewicz W., Michalczyk M. (2017). On the opposite trends of correlations between interaction energies and electrostatic potentials of chlorinated and methylated amine complexes stabilized by halogen bond. Theor. Chem. Acc..

[69] Fanfrlık J., Zierkiewicz W., Svec P., Rezac J., Michalczyk M., Ruzickova Z., Ruzicka A., Michalska D., Hobza P. (2017). Pnictogen bonding in pyrazine•PnX_5_ (Pn = P, As, Sb and X = F, Cl, Br) complexes. J. Mol. Model..

