# Supplementary material for: Comparison between Tetrel Bonded Complexes Stabilized by σ and π Hole Interactions
Source: Molecules. 2018 Jun 11;23(6):1416. doi: 10.3390/molecules23061416 (PMC6100375; doi:10.3390/molecules23061416)
Supplement: Supplementary file 1 [file molecules-23-01416-s001.pdf]

# Comparison Between Tetrel Bonded Complexes Stabilized by σ and π Hole Interactions

**Wiktor Zierkiewicz [1,*], Mariusz Michalczyk [1] and Steve Scheiner [2,*]**

[1] Faculty of Chemistry, Wrocław University of Science and Technology, Wybrzeże Wyspiańskiego 27, Wrocław 50370, Poland; mariusz.michalczyk@pwr.edu.pl

[2] Department of Chemistry and Biochemistry, Utah State University Logan, Utah 84322-0300, United States

* Correspondence: wiktor.zierkiewicz@pwr.edu.pl (W.Z.); steve.scheiner@usu.edu (S.S.); Tel.: +48-071-320-3455 (W.Z.); +1-435-797-7419 (S.S.)

Academic Editor: Steve Scheiner

Received: 30 May 2018; Accepted: 09 June 2018; Published: date

TABLE S1. NBO values of sum of the E(2) for LP(N)→σ*(T-X), (T= Si, Ge or Sn and X=H or F) orbital interaction and total charge transfer (CT) from $NH_3$ to $TH_{2-n}F_n$ in σ-hole bonded complexes obtained at the BLYP-D3(BJ)/def2-TVZPP level.

| Lewis acid[a] | ΣE(2) [kcal/mol] | CT [me] |
|---|---|---|
| $SiH_4$ | 3.30 | 19 |
| $GeH_4$ | 3.66 | 17 |
| $SnH_4$ | 6.41 | 30 |
| $SiH_3F$(a) | 15.14 | 83 |
| $SiH_3F$(b) | 3.44 | 20 |
| $GeH_3F$(a) | 17.07 | 78 |
| $GeH_3F$(b) | 4.21 | 19 |
| $SnH_3F$(a) | 22.75 | 93 |
| $SnH_3F$(b) | 16.94 | 69 |
| $SiH_2F_2$(a) | 24.40 | 107 |
| $SiH_2F_2$(b) | 6.65 | 33 |
| $GeH_2F_2$(a) | 26.30 | 96 |
| $GeH_2F_2$(b) | 38.62 | 123 |
| $SnH_2F_2$(a) | 34.82 | 117 |
| $SnH_2F_2$(b) | 54.87 | 168 |

[a]NBO analysis performed using DFT functional for the MP2 optimized geometries.

| | |
|---|---|
| $GeH_4$ | |

| | |
|---|---|
| 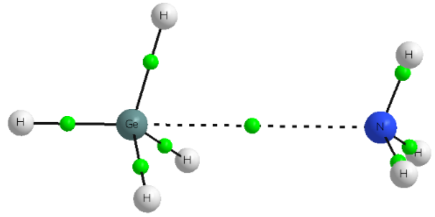 | |
| GeH$_3$F(a) | GeH$_3$F(b) |
| 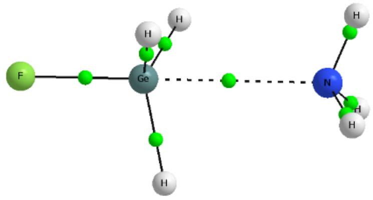 | 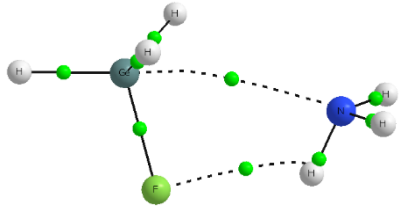 |
| GeH$_2$F$_2$(a) | GeH$_2$F$_2$(b) |
| 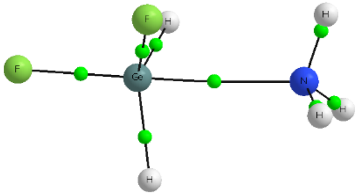 | 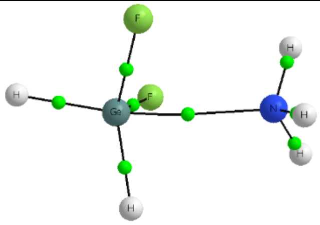 |

Figure S1. AIM diagrams showing the bond critical points (green dots) in Ge-containing complexes stabilized by σ-hole tetrel bonds.

TABLE S2. AIM data for σ-hole bonded complexes. Bond critical point (BCP) properties: electron density $\rho$, Laplacian of electron density $\nabla^2\rho$ (both in atomic units) and total electron energy (H, kcal $mol^{-1}$). Calculations were performed at the MP2/aug-cc-pVDZ-PP level.

| $NH_3\cdots$system | interaction | $\rho$ | $\nabla^2\rho$ | H |
|---|---|---|---|---|
| $SiH_4$ | Si···N | 0.008 | 0.025 | 0.51 |
| $GeH_4$ | Ge···N | 0.007 | 0.023 | 0.53 |
| $SnH_4$ | Sn···N | 0.011 | 0.033 | 0.40 |
| $SiH_3F$(a) | Si···N | 0.023 | 0.055 | -2.12 |
| $SiH_3F$(b) | Si···N | 0.009 | 0.028 | 0.46 |
| | F···N | 0.010 | 0.049 | 0.95 |
| $GeH_3F$(a) | Ge···N | 0.024 | 0.077 | -0.24 |
| $GeH_3F$(b) | Ge···N | 0.009 | 0.028 | 0.53 |
| | F···H | 0.013 | 0.053 | 0.40 |
| $SnH_3F$(a) | Sn···N | 0.027 | 0.089 | -0.55 |
| $SnH_3F$(b) | Sn···N | 0.021 | 0.063 | -0.21 |
| | F···N | 0.016 | 0.072 | 0.85 |
| $SiH_2F_2$(a) | Si···N | 0.032 | 0.055 | -5.07 |
| $SiH_2F_2$(b) | F···N | 0.013 | 0.046 | 0.36 |
| | F···N | 0.013 | 0.047 | 0.33 |
| | H···N | 0.014 | 0.037 | 0.12 |
| $GeH_2F_2$(a) | Ge···N | 0.033 | 0.099 | -2.14 |
| $GeH_2F_2$(b) | Ge···N | 0.041 | 0.114 | -4.42 |
| $SnH_2F_2$(a) | Sn···N | 0.037 | 0.123 | -1.52 |
| $SnH_2F_2$(b) | Sn···N | 0.050 | 0.182 | -2.83 |

TABLE S3. NBO values of sum of the E(2) for LP(N)→σ*(T-X), (T= Si, Ge or Sn and X=H or F) orbital interaction and total charge transfer (CT) from $NH_3$ to $TH_{2-n}F_n$=$CH_2$ in π-hole bonded complexes obtained at the BLYP-D3(BJ)/def2-TVZPP level.

| Lewis acid | ΣE(2) [kcal/mol] | CT [me] |
|---|---|---|
| $SiH_2$=$CH_2$ | 53.54 (42.89) | 163 |
| $GeH_2$=$CH_2$ | 32.81 (27.70) | 118 |
| $SnH_2$=$CH_2$ | 31.45 (23.88) | 113 |
| SiHF=$CH_2$ | 68.53 (44.69) | 194 |
| GeHF=$CH_2$ | 61.43 (37.38) | 175 |
| SnHF=$CH_2$ | 55.89 (26.78) | 171 |
| $SiF_2$=$CH_2$ | 75.96 (47.14) | 197 |
| $GeF_2$=$CH_2$ | 38.35 (24.04) | 193 |
| $SnF_2$=$CH_2$ | 63.06 (26.16) | 179 |

| $GeH_2=CH_2$(c) | $GeHF=CH_2$ (c) | $GeF_2=CH_2$(c) |
|---|---|---|
| 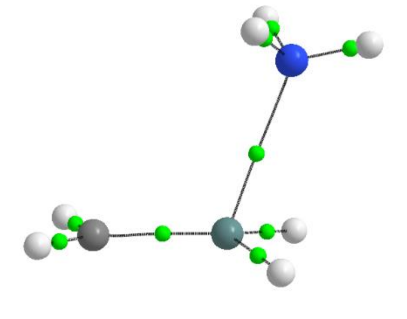 | 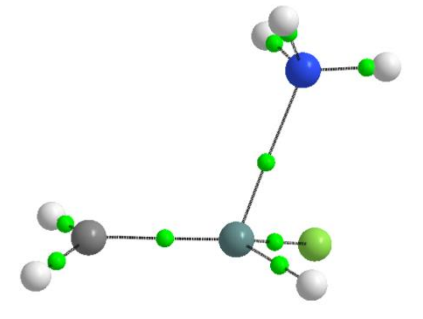 | 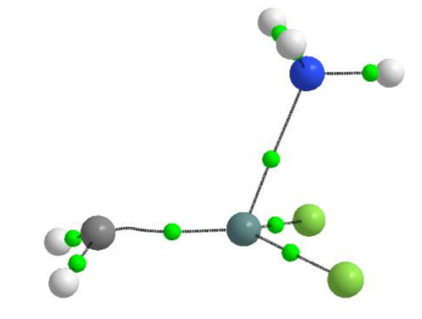 |

Figure S2. Bond critical points (green dots) in several Ge-containing complexes stabilized by π-hole tetrel bond.

TABLE S4. AIM data for π-hole bonded complexes. Bond critical point (BCP) properties: electron density $\rho$, Laplacian of electron density $\nabla^2\rho$ (both in atomic units) and total electron energy (H, kcal mol$^{-1}$). Calculations were performed at the MP2/aug-cc-pVDZ level.

| Lewis acid | $\rho$ | $\nabla^2\rho$ | H |
|---|---|---|---|
| $SiH_2=CH_2$ | 0.046 | 0.123 | -8.03 |
| $GeH_2=CH_2$ | 0.035 | 0.092 | -2.67 |
| $SnH_2=CH_2$ | 0.033 | 0.102 | -1.42 |
| $SiHF=CH_2$ | 0.057 | 0.226 | -7.65 |
| $GeHF=CH_2$ | 0.060 | 0.172 | -10.17 |
| $SnHF=CH_2$ | 0.052 | 0.194 | -2.96 |
| $SiF_2=CH_2$ | 0.064 | 0.279 | -7.90 |
| $GeF_2=CH_2$ | 0.074 | 0.225 | -14.07 |
| $SnF_2=CH_2$ | 0.059 | 0.230 | -3.87 |

Table S5.  Geometry and energetics for d complexes

| Lewis acid | $E_{int}$ | $E_{def}$ A | $E_{def}$ B | R(N···T) | ∠F-T···N | R(N···H1) | ∠T-H1···N | R(N···H2) | ∠C-H2···N | ∠ C–T-H1 |
|---|---|---|---|---|---|---|---|---|---|---|
| $SiHF{=}CH_2$ | -2.50 | 0.32 | 0.00 | 3.394 | 165.1 | 2.892 | 96.8 | 2.724 | 118.9 | 132.9 |
| $GeHF{=}CH_2$ | -4.23 | 1.02 | 0.00 | 3.058 | 169.2 | 2.808 | 84.5 | 2.662 | 108.7 | 143.4 |
| $SnHF{=}CH_2$ | -8.88 | 2.53 | 0.00 | 2.759 | 172.2 | 2.864 | 76.1 | 2.745 | 99.2 | 156.5 |

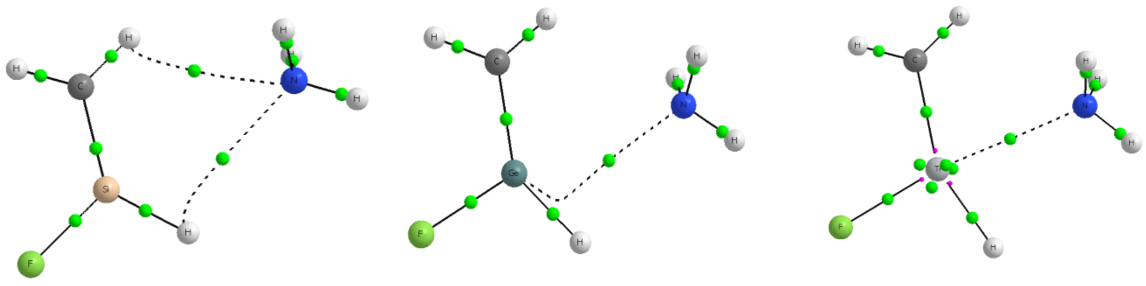

a) $SiHF{=}CH_2$ b) $GeHF{=}CH_2$ c) $SnHF{=}CH_2$

Figure S3.  AIM molecular diagram of $THF{=}CH_2/NH_3$ d dimers wherein the base occupies the d maximum of the MEP of the acid.

Table S6. NBO properties of d complexes

| Lewis acid | E(2) [kcal/mol] | CT [me] |
|---|---|---|
| $SiHF{=}CH_2$ | 1.35 (0.92)[a] | 11 |
| $GeHF{=}CH_2$ | 5.09 (3.75) | 30 |
| $SnHF{=}CH_2$ | 14.59 (7.77) | 73 |

[a]the largest value of the contribution in this donation

Table S7. AIM parameters of d complexes

| Lewis acid | interaction | $\rho$ | $\nabla^2\rho$ | H |
|---|---|---|---|---|
| $SiHF{=}CH_2$ | H···N | 0.007 | 0.025 | 0.60 |
| | H···N | 0.008 | 0.026 | 0.72 |
| $GeHF{=}CH_2$ | Ge···N | 0.012 | 0.035 | 0.56 |
| $SnHF{=}CH_2$ | Sn···N | 0.023 | 0.073 | -0.25 |

Table S8. EDA/BLYP-D3(BJ)/ZORA/TZ2P decomposition of the interaction energy of π-hole bonded complexes d into Pauli repulsion ($E_{Pauli}$), electrostatic ($E_{elec}$), orbital interaction ($E_{oi}$) and dispersion ($E_{disp}$) terms. All energies in kcal/mol. The relative values in percent express the contribution of each to the sum of all attractive terms.

| Lewis acid | $E_{int}$ | $E_{Pauli}$ | $E_{elec}$ | % | $E_{oi}$ | % | $E_{disp}$ | % |
|---|---|---|---|---|---|---|---|---|
| $SiHF{=}CH_2$ | -2.92 | 5.48 | -4.38 | 52 | -1.77 | 21 | -2.25 | 27 |
| $GeHF{=}CH_2$ | -4.55 | 11.16 | -9.22 | 59 | -3.67 | 23 | -2.81 | 18 |
| $SnHF{=}CH_2$ | -8.67 | 28.32 | -23.49 | 64 | -10.06 | 27 | -3.43 | 9 |

Table S9. Secondary minima for dimers of $NH_3$ with σ-hole donors. Data obtained at the MP2/aug-cc-pVDZ-PP level of theory. $E_{int}$ corrected for BSSE (in kcal/mol). Distances are in Å.

| $TH_nF_n$ System | $E_{int}$ | R(N···X) | structure |
|---|---|---|---|
| $SiH_4$ (b) | -0.50 | 3.600 | 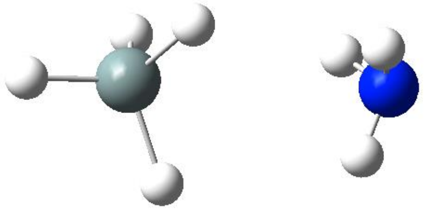 |
| $SiH_4$ (c) | -0.06 | 3.078 | 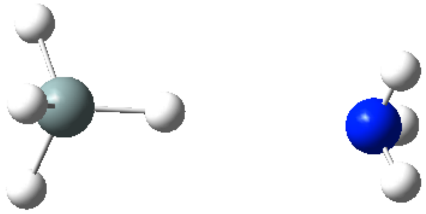 |
| $GeH_4$ (b) | -0.46 | 3.610 | 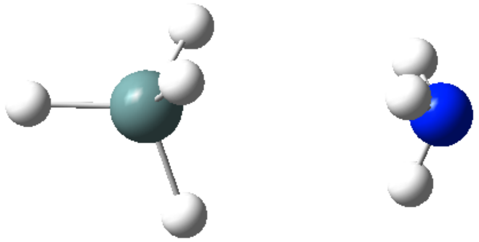 |
| $GeH_4$ (c) | -0.08 | 2.940 | 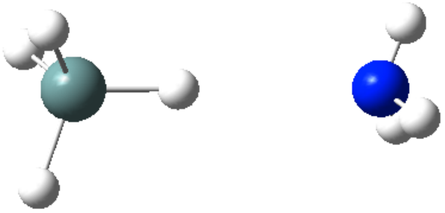 |
| $SiH_3F$(c) | -1.00 | 3.307 | 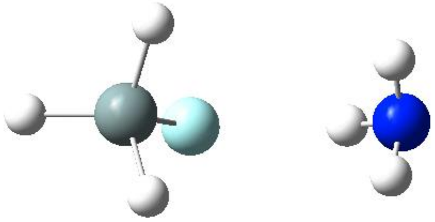 |
| $SnH_3F$(c) | -1.76 | 3.169 | 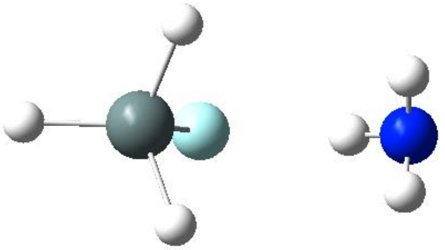 |

| | | | |
|---|---|---|---|
| $SiH_2F_2$(c) | -0.95 | 3.404 | 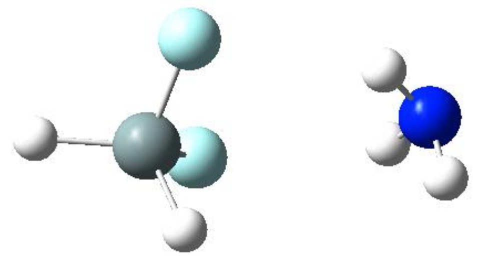 |
| $GeH_2F_2$(c) | -1.32 | 3.337 | 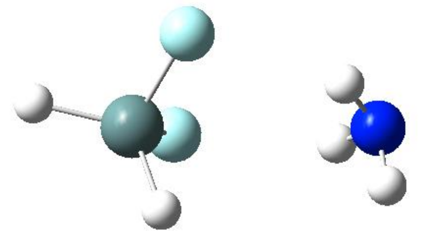 |
| $GeH_2F_2$(d) | -1.72 | 2.590 | 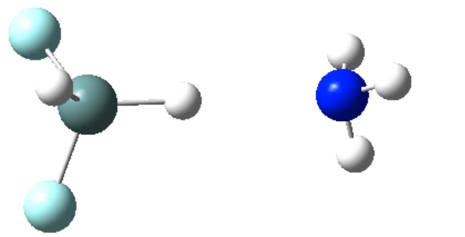 |
| $SnH_2F_2$(c) | -1.90 | 3.249 | 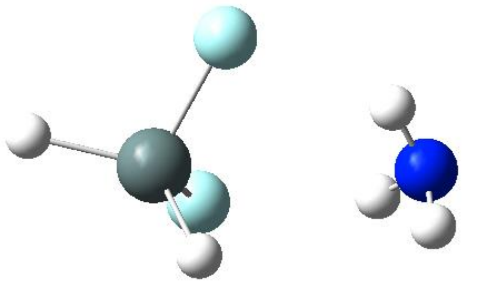 |
| $SnH_2F_2$(d) | -1.73 | 2.596 | 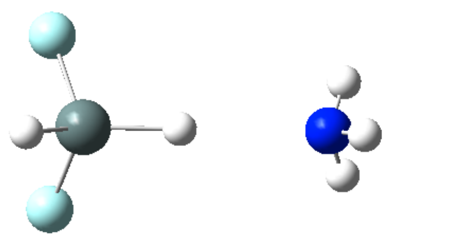 |

Table S10. Secondary minima for dimers of $NH_3$ with π-hole donors. Data obtained at the MP2/aug-cc-pVDZ-PP level of theory. $E_{int}$ corrected for BSSE (in kcal/mol). Distances are in Å.

| $TH_nF_n$ System | $E_{int}$ | R(N···X) | structure |
|---|---|---|---|
| $SiH_2$=$CH_2$ (b) | -1.65 | 2.500 | 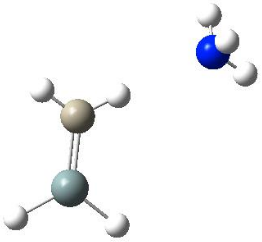 |
| $GeH_2$=$CH_2$ (b) | -1.16 | 2.473 | 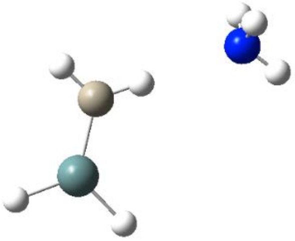 |
| SiHF=$CH_2$ (e) | -2.10 | 2.648 | 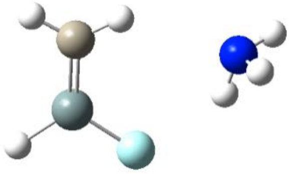 |
| GeHF=$CH_2$ (e) | -2.98 | 2.549 | 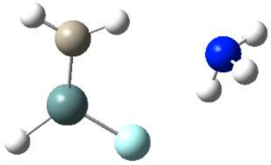 |
| GeHF=$CH_2$ (f) | -1.27 | 3.263 | 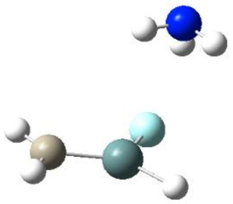 |
| SnHF=$CH_2$ (e) | -3.79 | 2.554 | 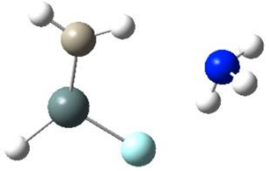 |
| SnHF=$CH_2$ (f) | -1.93 | 3.155 | 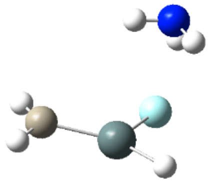 |

| | | | |
|---|---|---|---|
| $SiF_2$=$CH_2$ (b) | -1.88 | 2.405 | 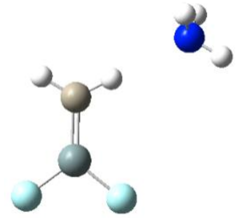 |
| $SiF_2$=$CH_2$ (c) | -2.32 | 2.612 | 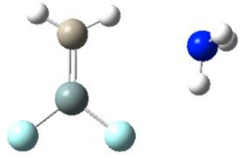 |
| $GeF_2$=$CH_2$ (b) | -3.64 | 2.600 | 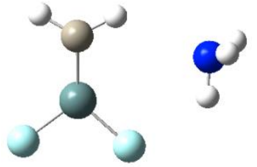 |
